# Factors associated with seasonal influenza self-diagnosis: a prospective observational study in Japan

**DOI:** 10.1038/s41533-020-0165-3

**Published:** 2020-03-23

**Authors:** Hiroki Maita, Tadashi Kobayashi, Takashi Akimoto, Fumihiko Matsuoka, Hiroshi Osawa, Hiroyuki Kato

**Affiliations:** 1 Development of Community Healthcare, Hirosaki University Graduate School of Medicine, Aomori, Japan; 2 General Medicine, Hirosaki University Graduate School of Medicine, Aomori, Japan; 3 Department of General Medicine, Hirosaki University School of Medicine & Hospital, Aomori, Japan; 4 Rokkasho Centre for Community and Family Medicine, Aomori, Japan

**Keywords:** Respiratory signs and symptoms, Population screening, Health care economics

## Abstract

This prospective observational study, conducted at a community clinic in Japan during the influenza season, from December 2017 to April 2018 aimed to investigate the accuracy of factors used for influenza self-diagnosis. Data were collected from pre-examination checklists issued to patients with suspected influenza and electronic medical records. Receiver operating characteristic (ROC) curve analysis was performed using a rapid influenza diagnostic test as the reference standard, and 2 × 2 contingency tables were analysed at each cut-off point. We analysed data from 290 patients (72.8% males, median age: 38 years, interquartile range: 26–50 years). The area under the ROC curve (AUC) for patients who were aware of other patients presumed to have influenza within close proximity was 0.74 (95% confidence interval (CI): 0.66–0.82). The AUCs for patients with a history of influenza, unvaccinated status, cough, or nasal discharge were 0.68 (95% CI: 0.60–0.75), 0.66 (95% CI: 0.59–0.73), 0.67 (95% CI: 0.59–0.75), and 0.70 (95% CI: 0.62–0.78), respectively. The sensitivity, specificity and positive likelihood ratio at a 90% cut-off point was 19.5% (95% CI: 13.5–26.6%), 94.1% (95% CI: 88.7–97.4%) and 3.31 (95% CI: 1.57–6.98). The sensitivity, specificity and negative likelihood ratio at a 10% cut-off point was 95.5% (95% CI: 90.9–98.2%), 9.6% (95% CI: 5.2–15.8%) and 0.48 (95% CI: 0.20–1.16). After multivariate logistic regression analysis, the AUC increased significantly from 0.77 (95% CI: 0.70–0.83) to 0.81 (95% CI: 0.76–0.86) when self-diagnosis-related information was added to basic clinical information. We identified factors that improve the accuracy and validity of influenza self-diagnosis. Appropriate self-diagnosis could contribute to the containment efforts during influenza epidemics and reduce its social and economic burden.

## Introduction

Influenza is an acute respiratory disease due to the influenza virus and is a common disease among patients presenting at outpatient clinics (14.6 million people/year in Japan^1^, 14.5 million people/year in the United States^2^) resulting in regional or seasonal epidemics (mainly in the winter^3^) and significant economic costs^4,5^.

In Japan, a rapid influenza diagnostic test (RIDT) has frequently been used to diagnose influenza. However, the RIDT is reported to have low sensitivity (Sn) (62.3%), with a specificity (Sp) of 98.2% in a meta-analysis^6^, and not be useful for early diagnosis, especially within 12 h of disease onset^7^. In many countries, the diagnosis of seasonal influenza is often performed clinically^8^. Early seasonal influenza is often misdiagnosed; however, if this could be overcome by accurate patient self-diagnosis, there could be potential to limit the spread of infection. Self-diagnosis could also enable viral containment if those diagnosed stay at home while they are infectious. With the development of information and communication technology, self-diagnosis and self-prescription using the information available on the internet have increased^9^, and interest in the accuracy of self-diagnosis has grown. Therefore, if a more accurate and faster self-diagnosis could be effectively made, it may help prevent the spread of infection, especially in places where medical resources are scarce^10^.

A retrospective study showed that the self-diagnosis index (extremely high or low probability) was useful for ruling in or ruling out an influenza diagnosis^11^. However, no studies have examined factors affecting the accuracy of influenza self-diagnosis, and no prospective observational studies have investigated the accuracy of the self-diagnosis index of influenza. Hence, in this study, we aimed to investigate the clinical factors improving the accuracy of the self-diagnosis index of seasonal influenza, to validate self-diagnosis accuracy using a prospective observational study design, and to analyse the synthetic clinical diagnostic value of adding self-diagnosis-related information to basic clinical information.

## Results

### Patient characteristics

**Fig. 1:**
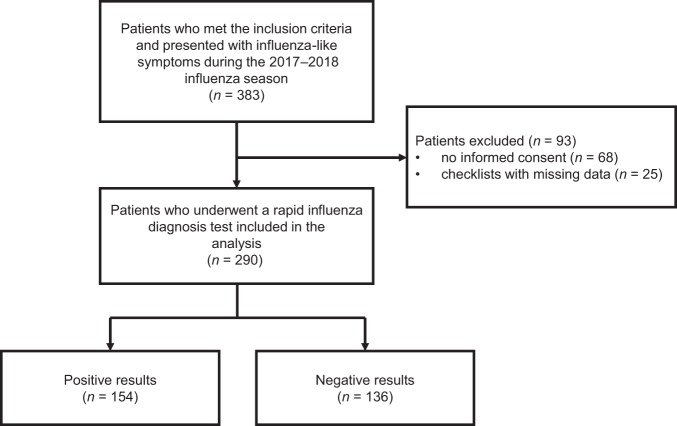
Flowchart of recruitment and study design.

In total, 383 patients met the inclusion criteria, and 290 patients (median age: 38 years; IQR 25%, 75%: 26, 50 years; male, 72.8%) were analysed (see Methods, Fig. 1). The study profile and demographics of the study population are summarised in Fig. 1 and Table 1, respectively.

**Table 1:** Baseline characteristics of the patients (*n* = 290).

Age (years) median (IQR 25%, 75%)	38 (26, 50)
12–17, *n* (%)	28 (9.7)
18–64, *n* (%)	251 (86.6)
≥65, *n* (%)	11 (3.8)
Sex, *n* (%)	
Male	211 (72.8)
Mean axillary temperature at the clinic, °C (SD)	37.8 (0.88)
Mean axillary temperature at home^a^, °C (SD)	37.8 (0.79)
Pulse rate, beats/min (SD)	98.7 (17.0)
Past medical history of influenza, *n* (%)	201 (69.3)
Awareness of other patients presumed to have influenza within close proximity, *n* (%)	142 (49.0)
Office	90 (31.0)
School	25 (8.6)
Home	23 (7.9)
Other	7 (2.4)
Influenza vaccination, *n* (%)	76 (26.2)
Patients taking medication prior to medical visits, *n* (%)	142 (49.0)
Symptoms, *n* (%)	
Patients who took their axillary temperature	241 (83.1)
Acute or sudden fever	147 (61.0)
Slow or no fever	94 (39.0)
Headache	166 (57.2)
Nasal discharge	153 (52.8)
Cough	193 (66.6)
Joint and muscle pain	141 (48.6)
Fatigue	206 (71.0)
Severity of current symptoms compared to those of a common cold, *n* (%)	
Severe	146 (50.3)
Similar	118 (40.7)
Mild	26 (9.0)
Duration (hours) from symptom onset to RIDT, median (IQR 25%, 75%)	39.8 (22.6, 64.0)
<12 h, *n* (%)	21 (7.2)
≥12 h, *n* (%)	269 (92.8)
Positive for RIDT, *n* (%)	154 (53.1)
Influenza A	79 (51.3)
Influenza B	75 (48.7)
Final clinical diagnosis (ICD-11), *n* (%)	
Influenza (1E30)	166 (57.2)
Acute upper respiratory infections (CA07)	109 (37.6)
Gastroenteritis (1A40)	7 (2.4)
Acute tonsillitis (CA03)	4 (1.4)
Acute bronchitis (CA42)	3 (1.0)
Allergic rhinitis (CA08)	2 (0.7)
Urinary tract infection (GC08)	1 (0.3)
Pharyngoconjunctival fever (1D84)	1 (0.3)
Significant clinical event that required hospitalisation, *n* (%)	1 (0.3)

*ICD-11* International Classification of Diseases 11th revision, *IQR* interquartile range, *RIDT* rapid influenza diagnostic test, *SD* standard deviation.

^a^*n* = 241.

### Area under the receiver operating characteristic (ROC) curve (AUC) of self-diagnosis for each category subgroup

Area under the ROC curve of self-diagnosis for each category subgroup with or without factors expected to affect the self-diagnosis accuracy can be found in Table 2. The AUC for the patient group that was aware of other patients presumed to have influenza located within close proximity to them was 0.74 (95% confidence interval (CI): 0.66–0.82), which was significantly higher than among patient groups who were unaware of other patients presumed to have influenza in close proximity to them. The AUCs of patient groups with a history of influenza infection, unvaccinated status, cough, or nasal discharge were 0.68 (95% CI: 0.60–0.75), 0.66 (95% CI: 0.59–0.73), 0.67 (95% CI: 0.59–0.75), or 0.70 (95% CI: 0.62–0.78), respectively; and these were relatively higher (difference in AUC > 0.05) than the patient groups without these factors. The AUC in the younger patient group (<18 years, *n* = 28) was 0.72 (95% CI: 0.54–0.91). In the older patient group (≥65 years, *n* = 11), the AUC was 0.38 (95% CI: 0.04–0.71). The AUC increased to 0.87 (95% CI: 0.75–0.98) in the patient group with all five factors present, apart from the age factor due to the small patient numbers.

**Table 2:** Area under the receiver operating characteristic curve of influenza self-diagnosis in each subgroup.

	AUC (95% CI)
Age (years)	
12–17, *n* (%)	0.72 (0.54–0.91)
18–64, *n* (%)	0.64 (0.58–0.71)
≥65, *n* (%)	0.38 (0.04–0.71)
Past medical history of influenza	
Yes	0.68 (0.60–0.75)
No	0.57 (0.45–0.68)
Awareness of other patients presumed to have influenza within close proximity	
Yes	0.74 (0.66–0.82)
No	0.54 (0.45–0.63)
Influenza vaccination	
Yes	0.60 (0.47–0.72)
No	0.66 (0.59–0.73)
Medication taken prior to medical visit	
Yes	0.67 (0.58–0.75)
No	0.62 (0.53–0.77)
Fever	
Acute or sudden	0.63 (0.55–0.72)
Slow or no fever	0.60 (0.49–0.71)
Headache	
Yes	0.63 (0.55–0.71)
No	0.64 (0.54–0.74)
Cough	
Yes	0.67 (0.59–0.75)
No	0.55 (0.40–0.69)
Nasal discharge	
Yes	0.70 (0.62–0.78)
No	0.59 (0.50–0.68)
Joint and muscle pain	
Yes	0.64 (0.55–0.73)
No	0.63 (0.55–0.72)
Fatigue	
Yes	0.65 (0.58–0.72)
No	0.63 (0.52–0.75)
Severity of current symptoms compared to those of a common cold	
Severe	0.62 (0.54–0.71)
Similar	0.67 (0.57–0.76)
Mild	0.58 (0.38–0.78)
Axillary temperature at the clinic (≥38.0 °C)	
Yes	0.59 (0.49–0.69)
No	0.63 (0.55–0.71)
Axillary temperature at the clinic (≥38.5 °C)	
Yes	0.61 (0.48–0.75)
No	0.63 (0.55–0.70)
Type of influenza	
A	0.65 (0.58–0.73)
B	0.63 (0.55–0.71)

*AUC* area under the curve, *CI* confidence interval.

### ROC curve analysis at multiple cut-off points

**Fig. 2:**
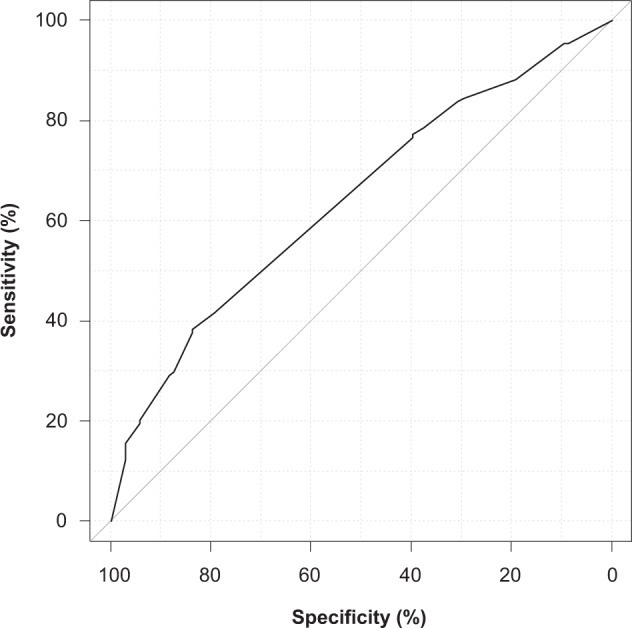
Receiver operating characteristic (ROC) curve of influenza self-diagnosis.

We performed ROC curve analysis at multiple cut-off points to verify the accuracy of influenza self-diagnosis. The AUC of influenza self-diagnosis (%) was 0.64 (95% CI: 0.58–0.70). The Sn, Sp, positive likelihood ratio (LR+), and negative likelihood ratio (LR−) were 38.3% (95% CI: 30.6–46.5), 83.8% (95% CI: 76.5–89.6), 2.37 (95% CI: 1.54–3.65), and 0.74 (95% CI: 0.64–0.85), respectively at the optimal cut-off point of 65% (Fig. 2). The LR− at the 10% cut-off point was 0.48 (95% CI: 0.20–1.16) and the LR+ at the 90% cut-off point was 3.31 (95% CI: 1.57–6.98). Other findings concerning Sn, Sp, LR+, and LR− at multiple cut-off points are listed in Table 3. In an additional analysis, 12 negative RIDT result cases were considered to be clinically diagnosed influenza to compensate for the low sensitivity of the RIDT as a reference standard, and the AUC was 0.64 (95% CI: 0.58–0.70), which did not show a significant difference.

**Table 3:** Receiver operating characteristic curve analysis of influenza self-diagnosis at various cut-off points.

Cut-off (%)	Sn (%), 95% CI	Sp (%), 95% CI	LR+, 95% CI	LR−, 95% CI	PPV, 95% CI	NPV, 95% CI	Youden index
10	95.5 (90.9–98.2)	9.6 (5.2–15.8)	1.06 (0.99–1.13)	0.48 (0.20–1.16)	0.54 (0.48–0.61)	0.65 (0.41–0.85)	0.05
20	88.3 (82.2–92.9)	19.1 (12.9–26.7)	1.09 (0.99–1.21)	0.61 (0.35–1.07)	0.55 (0.49–0.62)	0.59 (0.43–0.74)	0.07
30	83.8 (77.0–89.2)	30.9 (23.2–39.4)	1.21 (1.06–1.38)	0.53 (0.34–0.82)	0.58 (0.51–0.64)	0.63 (0.50–0.74)	0.15
40	78.6 (71.2–84.8)	37.5 (29.4–46.2)	1.26 (1.08–1.47)	0.57 (0.39–0.83)	0.59 (0.52–0.66)	0.61 (0.50–0.71)	0.16
50	76.6 (69.1–83.1)	39.7 (31.4–48.4)	1.27 (1.08–1.49)	0.59 (0.41–0.84)	0.59 (0.52–0.66)	0.60 (0.49–0.70)	0.16
60	41.6 (33.7–49.8)	79.4 (71.6–85.9)	2.02 (1.38–2.95)	0.74 (0.63–0.86)	0.70 (0.59–0.79)	0.55 (0.47–0.62)	0.21
70	37.7 (30.0–45.8)	83.8 (76.5–89.6)	2.33 (1.51–3.59)	0.74 (0.64–0.86)	0.73 (0.61–0.82)	0.54 (0.47–0.61)	0.22
80	29.2 (22.2–37.1)	88.2 (81.6–93.1)	2.48 (1.47–4.19)	0.80 (0.71–0.90)	0.74 (0.61–0.84)	0.52 (0.46–0.59)	0.17
90	19.5 (13.5–26.6)	94.1 (88.7–97.4)	3.31 (1.57–6.98)	0.86 (0.78–0.94)	0.79 (0.63–0.90)	0.51 (0.44–0.57)	0.14

*CI* confidence interval, *LR+* positive likelihood ratio, *LR−* negative likelihood ratio, *NPV* negative predictive value, *PPV* positive predictive value, *Sn* sensitivity, *Sp* specificity.

### The additional clinical diagnostic value of the self-diagnosis

**Fig. 3:**
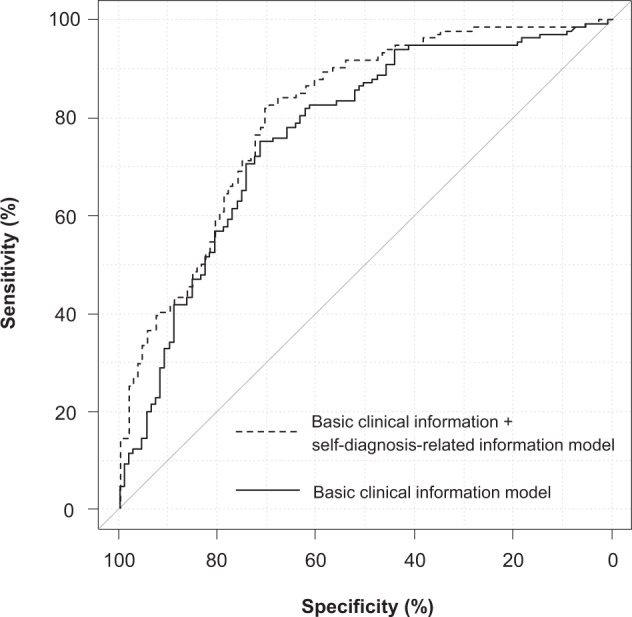
Receiver operating characteristic (ROC) curve for influenza diagnosis predicted according to a basic clinical information model vs. basic clinical information plus self-diagnosis-related information model. Basic clinical information included age, sex, headache, nasal discharge, cough, muscle and joint pain, fatigue, and axillary temperature at home. Self-diagnosis-related information included self-diagnosis of influenza (%), awareness of other patients presumed to have influenza within close proximity, past medical history of influenza infection, and influenza vaccination status.

Multivariate logistic regression analysis was performed to analyse the additional clinical diagnostic value of the self-diagnosis (Fig. 3). The AUC increased significantly from 0.77 (95% CI: 0.70–0.83) to 0.81 (95% CI: 0.76–0.86) (*P* = 0.03) when self-diagnosis-related information (self-diagnosis of influenza (%), awareness of other patients presumed to have influenza located within close proximity to the study patients, past medical history of influenza infection, and influenza vaccination status) was added to basic clinical information (age, sex, cough, headache, nasal discharge, fatigue, joint and muscle pain, and axillary temperature taken at home).

## Discussion

We conducted a prospective observational study and identified five important factors (awareness of other patients presumed to have influenza located within close proximity, history of influenza infection, unvaccinated status, cough, and nasal discharge) that increased the accuracy of influenza self-diagnosis. Accessing self-diagnosis-related information in addition to basic clinical information was useful for diagnosing influenza, especially when patients self-reported extremely high or low probability.

Past studies have reported that self-diagnosis, by dichotomous categories with yes and no responses, has low diagnostic accuracy (Sn, 45.7%; Sp, 58.1%)^12^. However, in a previous retrospective observational study, we showed that the self-diagnosis index was useful because we were able to evaluate the diagnostic accuracy at multiple cut-off points, especially when patients self-reported extremely high or low probability (LR+: 2.75 at 80% cut-off point, 95% CI: 0.75–10.07; LR−: 0.33 at 10% cut-off point, 95% CI: 0.12–0.96; AUC: 0.63, 95% CI: 0.53–0.73)^11^. In the current prospective observational study, we confirmed the usefulness of the self-diagnosis index (LR+: 3.31 at 90% cut-off point, 95% CI: 1.57–6.98; LR−: 0.48 at 10% cut-off point, 95% CI: 0.20–1.16; AUC: 0.64, 95% CI: 0.58–0.70) (Table 3), which is consistent with our previous results.

Our study had several strengths. For the first time, to our knowledge, we identified five factors that can improve the accuracy of self-diagnosis. Factors such as history of influenza infection, unvaccinated status, cough, or nasal discharge tended to improve the accuracy of self-diagnosis, indicating that the presence of subjective symptoms can render self-diagnosis more accurate. Although there has been no direct research on whether influenza vaccinations reduce the symptoms of influenza, their effectiveness in reducing the incidence of influenza is clear^13^ and the alleviation of symptoms can negatively affect the accuracy of self-diagnosis. Information regarding a history of influenza infection may have helped patients to distinguish between the common cold and influenza through enabling a comparison between past and current symptoms. Additionally, we showed that patients’ awareness of other patients presumed to have influenza within close proximity significantly improved the accuracy of self-diagnosis. The shape of the graph showing medical visits for influenza at the research clinic was similar to that at sentinel sites in the research area and we consider that our data reflected the epidemic situation in the area (Supplementary Fig. S1). The peak in the number of patients who were aware of patients presumed to have influenza within close proximity to them overlapped with a peak in the number of patients with influenza at the research clinic (Supplementary Fig. S2); therefore, the more patients who visited the clinic, the more self-diagnosis was considered to be clinically useful because the proportion of patients with high diagnostic accuracy of self-diagnosis increased in line with the epidemic. In recent years, several studies have pointed out that local, influenza-related Google search frequency corresponded to local influenza epidemic status^14,15^. The search frequency for “influenza” in the research area (Aomori Prefecture) during the study period also corresponded to the epidemic situation in the area (Supplementary Fig. S3). As described, there are several methods to confirm a local influenza outbreak, such as direct observation of the area, information derived from a local medical institution, and information provided on the internet. We showed that confirmation of a local influenza outbreak by patients’ direct observations (confirmation at places such as their workplace, school, or home) was the most reliable factor for improving the accuracy of influenza self-diagnosis. Second, this was a prospective observational study and was larger than our previous retrospective study^11^; therefore, the quality of the collected data was improved, and selection bias was reduced. Moreover, the results of this study increased the reliability of influenza diagnostic accuracy and were consistent with the results and conclusions of previous research. Third, using multivariate logistic regression analysis, this study showed that self-diagnosis-related information was additionally useful for influenza diagnosis in general practice. For diagnostic clinical information to be useful, it is important that it improve overall diagnostic accuracy when added to existing basic clinical information, rather than being used as a form of independent individual information. Finally, this study involved recent baseline data on the self-diagnosis index of influenza. In recent years, many patients have obtained health information on the internet prior to medical visits^16,17^, which may affect self-diagnosis and treatment behaviour. The cultural background situation in relation to the development of technology is changing rapidly; therefore, additional studies undertaken at appropriate times are necessary.

Our study also had a few limitations. First, the interpretation of our results may differ depending on the age group. In the ≥65 years group, the accuracy of self-diagnosis was extremely poor (Table 2). There were only 11 cases analysed in this group and it was difficult to validate the results statistically. Therefore, the results of this study cannot be extended to elderly populations. Second, we needed to select a more accurate reference standard. In Japan, especially in primary care clinics, it is usually difficult to perform polymerase chain reaction tests or obtain viral cultures, which are highly accurate reference standards for influenza diagnosis. Future studies are needed where more accurate tests can be easily used for influenza diagnosis in primary care settings. Third, patient background factors, such as education levels and different cultures, should be considered. In Japan, the concept of percentages is taught to fifth graders (10- and 11-year olds). Therefore, it may be necessary to change the types of questions used, depending on patients’ age and the education system in each country involved. Fourth, the answers of the pre-examination checklist about influenza were based on participants’ recall and knowledge of encountering someone presumed to have influenza, and we could not check their reliability. However, even if their information affected the accuracy of the self-diagnosis, we believe that it was useful as a daily medical care tool. Finally, a prospective study with the main objective of validating the factors suggested to be effective in this study is required. To use influenza self-diagnosis more effectively, additive disease factors (e.g. strains of influenza), patient factors (e.g. more detailed symptoms, including patients with no symptoms, cognitive function, sources of information, internet, and smartphone use), and environmental factors (e.g. residential areas (such as urban areas), remote areas, or other countries) that were not included in this study should be analysed.

Our results have practical significance in the following two settings in terms of influenza epidemic suppression and social burden reduction. First, in this study, we identified factors for improving self-diagnosis that can be applied to acquiring medical history information in telemedicine as well as in ordinary clinical examinations. In this study, we showed that adding self-diagnosis-related information to basic clinical information significantly improved diagnostic accuracy (Fig. 3), which indicates that the accuracy of influenza diagnosis can be improved without direct face-to-face communication and can be used in telemedicine. In recent years, telemedicine has been reported to be useful not only for the management of chronic diseases^18^ and for improvement in the quality of emergency care^19^, but also for initial treatment of epidemic diseases^20^. Minimising clinical visits of outpatients with mild symptoms involving a low complication risk is important in controlling influenza transmission because visits to a medical institution may result in potential exposure to an influenza virus^21^. Second, self-diagnosis can contribute to efficient screening by applying different cut-off points (e.g. high Sn at 10% cut-off point, high Sp at 90% cut-off point). In general, self-diagnosis is considered to be useful in situations where medical care is unavailable^22^, in avoiding burdening the public health system^22^, or where early diagnosis has a significant effect on disease prognosis^10^. In situations where infection control is a major social issue (e.g. pandemic influenza), effective action can be taken based on patient risk without assessment at a medical institution.

In conclusion, we identified factors that increased the accuracy of influenza self-diagnosis such as awareness of proximity to others presumed to have influenza. Accessing self-diagnosis-related information in addition to basic clinical information was useful for diagnosing influenza, especially when patients self-reported extremely high or low probability. Appropriate self-diagnosis could contribute to the containment efforts during influenza epidemics and reduce its social and economic burden.

## Methods

### Study design and ethical considerations

We conducted a prospective observational study at a community clinic in Rokkasyo-mura, Aomori, Japan (Rokkasho Centre for Community and Family Medicine) and collected data on factors affecting the accuracy of influenza self-diagnosis, then verified the self-diagnostic data for accuracy and assessed the additional diagnostic value of self-diagnosis. This study was approved by the Medical Ethics Committee of Hirosaki University (approval number: 2017-1100). All data were fully anonymised at the time of data collection, and written informed consent was obtained from all patients included in the study.

### Patients

We invited all patients who were clinically suspected of seasonal influenza (e.g. upper respiratory tract symptoms and fever) to complete a pre-examination checklist during the influenza season, from December 2017 to April 2018. Patients who met all three of the following criteria were included in the study: (1) aged ≥ 12 years, (2) had completed a pre-examination checklist prior to a doctor’s assessment, and (3) had undertaken an RIDT. Patients were excluded from the study if they: (1) did not complete all the items on the pre-examination checklist, or (2) did not provide written consent.

### Data collection

Data were collected using pre-examination checklists (Supplementary Figs S4, S5) and electronic medical records. Items included in both data sources reflected not only clinical symptoms but also factors that were presumed to be important for self-diagnosis, as reported in past studies^11,23–27^, or that were clinically important. Items on the pre-examination checklist included: a past medical history of influenza infection, influenza vaccination status, whether medication had been taken prior to medical visits, awareness of other patients with influenza within close proximity to the study patients (at work, at school, at home, or at another location in close proximity), clinical symptoms (headache, nasal discharge, cough, joint and muscle pain, fatigue, history of fever (acute/sudden or slow)), axillary temperature at home, time of symptom onset, severity of current symptoms (compared to having a common cold), and self-diagnosis presented as a percentage (%). Items collected from patients’ medical records were as follows: age, sex, clinical signs (axillary temperature, pulse rate at clinic), time of RIDT, results of RIDT, and a physician’s final diagnosis.

The self-diagnosis index is a continuous measure, which offers researchers the ability to query the degree to which participants were certain of their diagnosis. This is in juxtaposition to the dichotomous categories of yes and no responses, which do not accommodate the absence of certainty. We used the percentage as a continuous variable for the self-diagnosis index and asked the patient to provide an estimate of the possibility that they were suffering from influenza on the pre-examination checklist.

### Diagnosis of influenza

As a reference standard for diagnosing influenza infection, a rapid diagnostic kit (Prime Check Flu, Alfresa Co., Ltd., Japan) was used and laboratory diagnosis was determined by a clinical laboratory technician independent of the physicians attending the patients and independent of the nurses who conducted the pre-examination.

### Statistical analysis

ROC curve analysis was performed to estimate the AUC and the optimal cut-off point. The AUC was determined to evaluate the discriminatory power of the self-diagnosis under various conditions^28^, such as the presence or absence of clinical factors. To calculate Sn, Sp, and the likelihood ratio at each cut-off point, 2 × 2 contingency tables were analysed. To validate the additional contribution of self-diagnosis in predicting the influenza diagnosis, we added self-diagnosis-related information to a multivariate logistic regression analysis including basic clinical signs. We expected the rate of positive influenza testing about 50% with reference to our previous study^11^, and estimated a sample size of at least 240 based on the minimum 10 events per variable rule for logistic regression analysis^29^. All statistical analyses were performed with EZR version 1.37 (Saitama Medical Centre, Jichi Medical University, Saitama, Japan), which is a modified version of R Commander that is designed to add statistical functions frequently used in biostatistics^30^.

### Reporting summary

Further information on research design is available in the Nature Research Reporting Summary linked to this article.

## Supplementary information

Supplementary Information

Reporting Summary

## Data Availability

The data generated and/or analysed during the current study are mostly included in this published article. Additional data are available from the corresponding author upon reasonable request.
