# Supplementary material for: Factors associated with seasonal influenza self-diagnosis: a prospective observational study in Japan
Source: NPJ Prim Care Respir Med. 2020 Mar 23;30:9. doi: 10.1038/s41533-020-0165-3 (PMC7090047; doi:10.1038/s41533-020-0165-3)
Supplement: Supplementary file 1 — Supplementary Information [file 41533_2020_165_MOESM1_ESM.pdf]

Supplementary Figure 1.

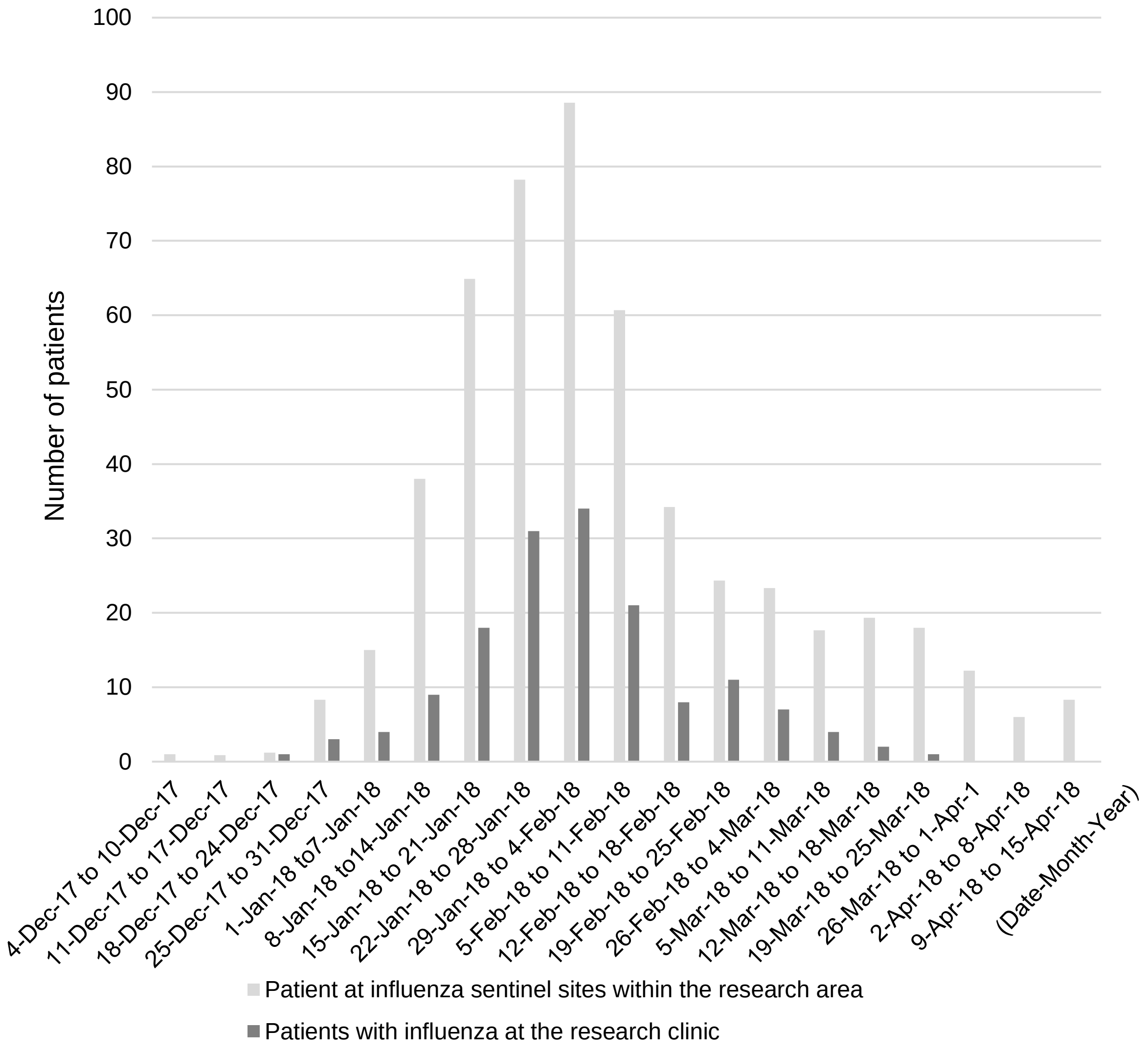

Supplementary Figure 1. Comparison of influenza at local sentinel sites vs. our research clinic. Patients with influenza at sentinel sites in the research area (light grey) and patients with influenza at the research clinic (dark grey).

Supplementary Figure 2.

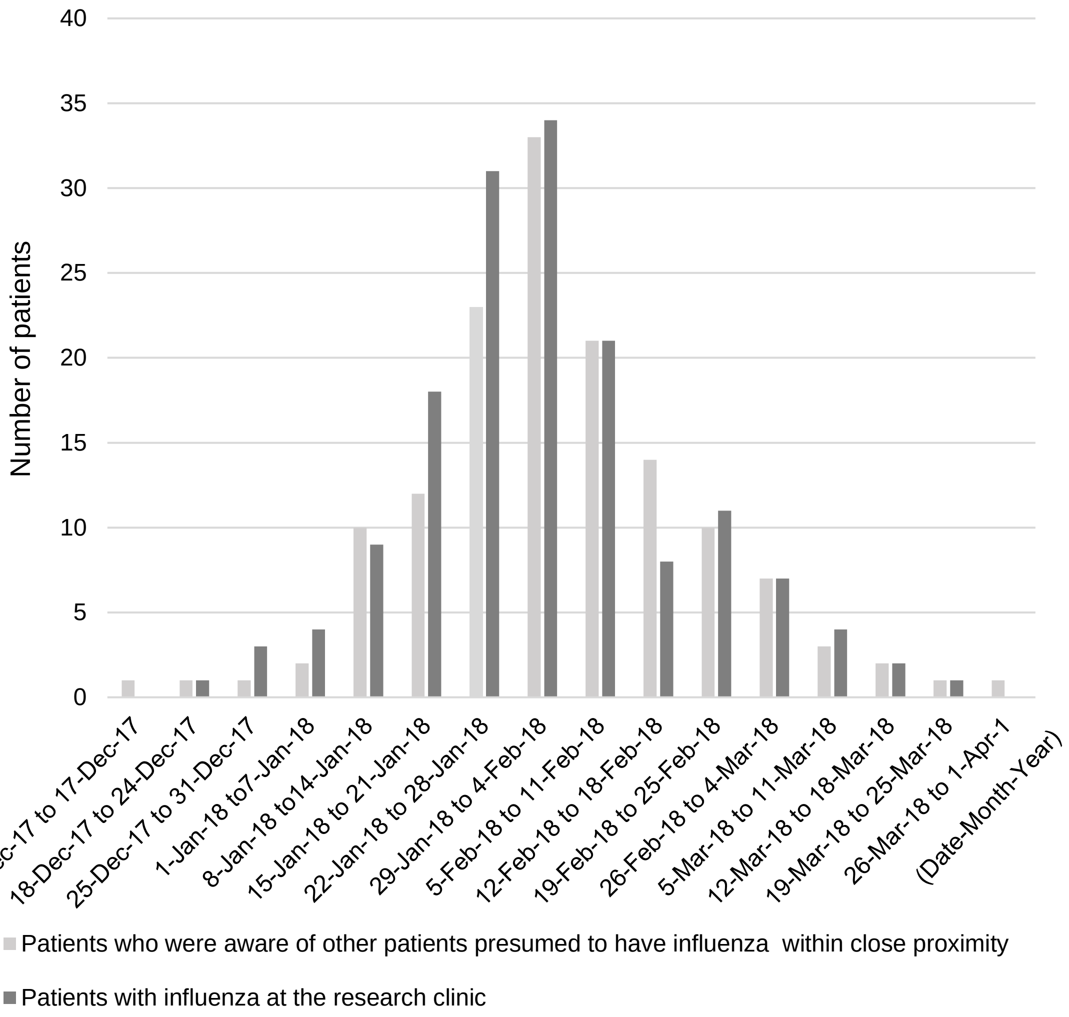

Supplementary Figure 2. Influenza at our research clinic compared to patients who were aware of others with influenza in close proximity. Patients who were aware of other patients presumed to have influenza within close proximity (light grey) and patients with influenza at the research clinic (dark grey).

Supplementary Figure 3.

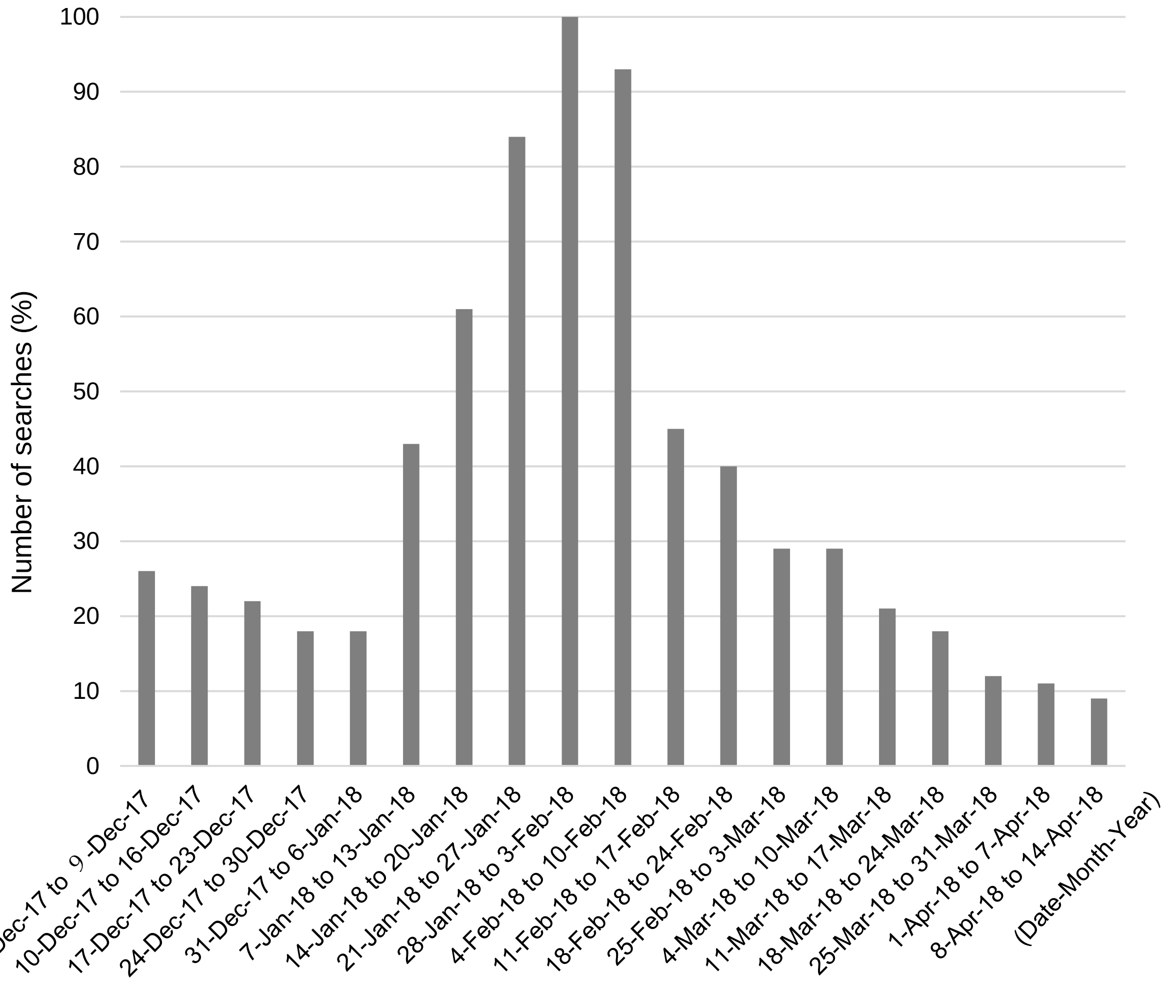

Supplementary Figure 3. The internet search frequency for “influenza” in the research area (Aomori Prefecture) during the study period analyzed using Google trends. The resulting numbers were scaled on a range of 0 to 100% based on the word "influenza" as a proportion of all searches on all topics.

Supplementary Figure 4.

インフルエンザ疑い患者に対するチェック項目

1. **今までにインフルエンザに罹ったことはありますか?** □ はい □ いいえ

2. **身の回りにインフルエンザの患者がいますか?** □ はい □ いいえ

   「はい」方へ。それはどこですか。

   □ 職場 □ 学校 □ 家族 □ その他（ ）

3. **今年度、インフルエンザの予防接種を受けていますか？**

   □ はい □ いいえ

4. **受診前に、体温を測りましたか？** □ はい □ いいえ

   「はい」の方へ。何度でしたか。 ________℃

   「はい」の方へ。突然または急に熱が上がりましたか。 □ はい □ いいえ

5. **受診前に、薬を飲みましたか？** □ はい □ いいえ

   「はい」の方へ。なんというお薬を飲みましたか。（ ）

6. **以下の症状のうち、当てはまるもの全て答えてください。**

   □ 頭痛 □ 鼻汁 □ 咳嗽

   □ 体の痛み □ 体のだるさ □ その他（ ）

   いつそれらの症状が始まりましたか？

   月 日 時 分頃

7. **今回の症状は、いつもカゼ（普通のカゼ）よりもつらいですか？**

   □ つらい □ 同じくらい □ つらくない

8. **インフルエンザの可能性はどれくらいだと感じていますか？**

   ________% (0 – 100%)

氏名 ________ **年齢** **歳** **性別** **男 / 女**

Supplementary Figure 4. Pre-examination checklist (Japanese version).

Supplementary Figure 5.

PRE-EXAMINATION CHECKLIST

1. **Have you ever had influenza?** ☐ Yes ☐ No
2. **Do you know of any current patients with influenza within close proximity to you?** ☐ Yes ☐ No

   If "Yes", please answer where the places are.

   ☐ Office ☐ School ☐ Home ☐ Other ( )
3. **Have you had an influenza vaccination this season?** ☐ Yes ☐ No
4. **Did you take your body temperature prior to this visit?** ☐ Yes ☐ No

   If "Yes", please indicate your temperature reading. ________ ℃

   If "Yes", did your temperature rise acutely or suddenly? ☐ Yes ☐ No
5. **Did you take any medication for your symptoms prior to your visit?** ☐ Yes ☐ No

   If "Yes", please provide the name of the medication. ( )
6. **Please indicate below which of the following symptoms you have.**

   ☐ Headache ☐ Runny nose ☐ Cough

   ☐ Muscle and joint pain ☐ Fatigue ☐ Other ( )

   When did the first symptom start?

   Date ____ (dd) ____ (mm) Time ___ : ___ a.m. / p.m.
7. **How severe are symptoms (compared to a common cold)?**

   ☐ Severe ☐ Similar ☐ Mild
8. **How certain are you of the possibility that you may have influenza?**

   ________ % (0 – 100%)

NAME ________________ **AGE** ______ **SEX** **M / F**

Supplementary Figure 5. Pre-examination checklist (English translation version).
